# Effects of aflatoxin B_1_ on reproductive performance of farmed Nile tilapia

**DOI:** 10.1080/23144599.2019.1678315

**Published:** 2019-10-19

**Authors:** Esther Marijani, Harrison Charo-Karisa, Gbemenou Joselin Benoit Gnonlonfin, Emmanuel Kigadye, Sheila Okoth

**Affiliations:** a Open University of Tanzania, Department of Food and Nutrition, Dar es Salaam, Tanzania; b WorldFish, Fish 4Africa Innovation Hub, Abbassa, Egypt; c Economic Community of West African States (ECOWAS), Asokoro, Nigeria; d School of Biological Science, University of Nairobi, Nairobi, Kenya

**Keywords:** Aflatoxin B_1_, growth, fecundity, milt, testosterone, 17β-oestradiol

## Abstract

This study evaluated the effect of dietary aflatoxin B_1_ (AFB_1_) on growth, milt and egg quality in matured Nile tilapia (*Oreochromis niloticus*). Triplicate groups of Nile tilapia (initial body weight 24.1 ± 2.6 g) were fed with either of four diets (Diets 1 to 4) designed to contain 0, 20, 200 and 2000 μg AFB_1_ kg^−1^ diets for 24 weeks. After 24 weeks of AFB_1_ exposure, growth was significantly (*P <*0.05) different between the control and the AFB_1_ exposed treatments in both sexes. No significant differences were observed in 17β-oestradiol, absolute fecundity, oocytes volume and diameters between AFB_1_ exposure groups and the control group. However, we observed a significant reduction in relative fecundity and gonad somatic index (GSI) in females fed 2000 μg AFB_1_ kg^−1^ diet. On the other hand, we observed significant differences (*P <*0.05) in gonadosomatic index (GSI), testosterone, milt count and motility between males in the control group and AFB_1_ treatments. We conclude that rearing Nile tilapia with aflatoxin-contaminated diets for a prolonged period affects milt quality, fecundity (at higher doses) and growth performance. This implies that for optimal seed production, provision of aflatoxin free diets should be part of the management practices in Nile tilapia hatcheries.

## 1. Introduction

Nile tilapia (*Oreochromis niloticus*) is East Africa’s most cultured fish species and is farmed under a range of semi-intensive and intensive systems [1]. It is known for rapid growth, tolerance to high stocking densities and poor water quality, high reproductive rates and low susceptibility to disease. However, obtaining good quality locally made feed is the largest constraint for semi-intensive and intensive aquaculture of the species in the region [1]. To lower costs, the use of plant-based ingredients in aquafeeds is increasing. However, plant-based ingredients are very susceptible to aflatoxin contamination at different stages of the agricultural chain such as pre-harvest, harvest and post-harvest handling. Thus, feeds are the possible source of aflatoxin exposure of farmed Nile tilapia [2].

Aflatoxins (AF) are mycotoxins produced mostly by the fungi *Aspergillus flavus* and *A. parasiticus*, which grow on numerous feedstuffs when environmental conditions are favourable [3]. There are four common types of aflatoxin–aflatoxins B_1_, B_2_, G_1_ and G_2_ and among them; AFB_1_ is the most potent one to human and animals [4]. Aflatoxins are some of the most dangerous mycotoxins that are characterized and famous for their high toxicity to both animals and humans. Aflatoxins contamination has been reported to cause a reduction in fish productivity as well as fish mortality [5]. They cause anaemia, haemorrhaging, liver impairment, decreased weight, increased vulnerability to secondary infectious diseases and increased mortality resulting in significant problems in aquaculture [5].

Aflatoxins can affect reproductive performance directly generating hormonal dysfunction and inducing cell toxicity. When hormonal functions are disrupted in fish, it may affect their sexual maturation, gamete production and transport, sexual behaviour, fertility, gestation, modifications in other functions that are dependent on the integrity of the reproductive system [6].

Aflatoxins have also been reported to reduce egg size and volume in gibel carp and reduce hatchability, egg production and quality in poultry [7–9]. Aflatoxins reduce sperm concentration, and impair survival of spermatozoa in boars, and decrease semen volumes and testis weights in juvenile Japanese quail [10–13]. Egg and milt quality of fish are critical in fertilization success [14]. Also, milt quality is a very important variable in aquaculture broodstock management and influences egg fertilization rates [14]. However, there is limited information about the effects of aflatoxins in egg and milt quality of Nile tilapia reared in ponds.

Many studies have documented the association between aflatoxins and growth, primary liver cancer, haematocrit and humoral immune response in Nile tilapia, but little is known about the relationship between this toxin and egg and milt quality in this species. Here, we investigate the toxic effects of AFB_1_ on the reproductive hormones, egg and milt quality of Nile tilapia reared in ponds for 24 weeks. Considering that toxic effect of AFB_1_ depends on the dose in the feeds as well as sex and the age of animal [15], we also examined whether males and females of Nile tilapia exhibit differences in their growth response to dietary AFB_1_ contamination.

## 2. Materials and methods

### 2.1. Experimental system and animals

The experiment was carried out in 12 cages (1 m^3^ each) placed in a 40 × 20 m pond at the National Aquaculture Research and Development Centre (NARDTC), Sagana, Kenya. Throughout the experiment water quality parameters were maintained as follows: temperature from 24.2°C to 26.8°C; pH from 7.5 to 7.65; dissolved oxygen, 4.6–6.3 mg L^−1^; total alkalinity, 146.65–225.95 mg L^−1^; and salinity, 0.03–0.05 ppt.

Nile tilapia fingerlings (24.1 ± 2.6 g) were obtained from NARDTC, Sagana. Fishes were first acclimated to their cages conditions and fed with the control diet for 15 days before dietary treatments.

### 2.2. Experimental design and feeding trials

A pure crystalline powder of AFB_1_ was obtained from Libios Chemical Company Ltd, Rue Edmond Michelet, France. The AFB_1_ (5 mg) was dissolved in 100 mL of pure methanol to form a stock solution containing 0.05 mg AFB_1_ mL^−1^ of methanol. Four experimental diets, Diet 1, 2, 3 and 4 were formulated and designed to, respectively, contain 0, 20, 200 and 2000 μg AFB_1_ kg^−1^ of feed and the determined levels were 0.68, 22.5, 235.4 and 2046.5 μg AFB_1_ kg^−1,^ respectively. The formulation of the basal diet is shown in Table 1. Diets were made into pellets (5 mm, diameter), oven-dried at 60°C and stored at −20°C until fed. The given toxin levels and control feeds were cross-checked twice a month through Enzyme-Linked Immunosorbent Assay (ELISA) (BioTek Instruments, Inc., VT, USA).

**Table 1.** Formulation and chemical composition of basal diet (in dry weight).

Ingredients	Content (g/100 g diet)
Soybean meal	30
Shrimp meal	17.9
Wheat bran	5
Sunflower seedcake	40.57
Sunflower oil	2
Cassava flour	1.5
Vitamin premix^a^	1.06
Starch	1.97
Chemical analysis (% or kJ g^−a^ in dry matter))	
Crude protein	30.83
Ether extract	5.93
Ash	5.93
NFE	51.56
GE (Kcal/g)	441.89

NFE – Nitrogen free extract

GE – Gross energy

^a^Vitamin premix (mg kg^−1^ diet): vitamin A, 18 M.I.U.; vitamin D3, 4 M.I.U.; vitamin E, 6.5g; vitamin B2, 3.5 g; vitamin K3, 2 g; nicotinic acid, 17 g; pantonthenic acid, 7 g; folic acid, 0.4 g; vitamin B1, 1.5 g; vitamin B6, 2.5 g; vitamin C, 12 g; magnesium, 6 g; potassium, 7.5 g; sodium, 20 g; citric acid, 18 g

### 2.3. Experimental feeding regime

A total of 300 Nile tilapia fingerlings were divided into 12 equal groups of 25 fish and placed in cages. Three replicates of 25 fish each were randomly assigned to each treatment and fed on experimental diets following acclimatization. Fish were fed to apparent satiation twice a day at 0900 and 1600 h. Pellets were slowly fed to the fish to minimize feed wastage. The trial lasted for 24 weeks. At the end of the experiment, fish from each cage was sampled randomly after 1 day of food deprivation.

### 2.4. Growth performance

Each month, all the fish in each experimental unit were sampled and measured to determine number, weight and length. At the end of the experiment, 18 fish (9 males and 9 females) from each dietary treatment were sampled randomly after a day of food deprivation. Average weight gain (AWG), average daily gain (AVG) and survival rate (SR%) were calculated according to Tola et al. [16].

### 2.5. Fecundity

The females were weighed and dissected to remove ovaries which were also weighed. Gonadosomatic index (GSI) was calculated as ovary weight divided by body weight multiplied by 100. Egg numbers in the ovaries were counted to determine absolute fecundity (AF) and relative fecundity (RF) was calculated by dividing the total egg number by total body weight [17]. Egg weight (dry and wet basis) was determined using 50-count egg samples: a sample of 50 eggs was weighed and oven-dried at 70°C for 48 h. Eggs diameter was measured using a microscope eye-piece graticule for length (L) and width (H). Egg volume was calculated by the formula: V = π LH^2^/6 [18], where: V = egg volume (mm^3^), L = long axis (mm), H = short axis (mm), π = 3.14.

### 2.6. Milt quality

At the end of the experiment, males were selected randomly, weighed and dissected, testes removed and gonad somatic index (GSI) determined. Milt samples from males were taken by hand stripping. Milt motility was estimated by microscopic observation of milt diluted 100-fold in a distilled water solution. Forward motility was assessed according to Tekin [19]. Milt volume was determined by measuring with a pipette and expressed in ml. Spermatozoa concentration was determined using haemocytometer and expressed as number of cells x 10^9^ cell ml^−1^ [19].

### 2.7. Sex-steroid hormone measurements

The sex hormones (testosterone and 17β-oestradiol) were analysed using Enzyme-Linked Immunosorbent Assay (ELISA) (BioTek Instruments, Inc., VT, USA). This was done using commercially available ELISA kits from Human Chemical Company, Germany according to supplier’s instructions. (Intra-assay coefficients of variation were 3.5% and 3.8% while inter-assay coefficients of variation were 11.8% and 9.8% for testosterone and 17β-oestradiol, respectively.

### 2.8. Data analysis

Data analyses were performed using a two-way ANOVA (randomized block) (Genstat 4.0) that examined the main effects of AFB_1_ contamination, duration and their interactions and means were tested by Bonferroni test at the 5% level of significance using GenStat 4.0. For the effect of AFB_1_ contamination in sex hormones, egg and milt quality, one-way ANOVA followed by Bonferroni test at the 5% level of significance procedure was followed using GenStat 4.0.

## 3. Results

### 3.1. Growth performance

Aflatoxin significantly (*P* <0.05) depressed growth rate, average weight gain and average daily gain in both sexes (Table 2). Mean final weight was higher in control group of female Nile tilapia (91.67 g) compared to exposure groups (79.16, 68.86, 59.25 gfor diet 2, 3 and 4, respectively). Mean final weight of male Nile tilapia was also affected by AFB_1_ whereas the final mean weight of exposure groups was lower than that of control group (Table 2). Aflatoxin adversely affected average weight gain *(P <*0.05) in both sexes. AWG was decreased with increased AFB_1_ concentrations. As shown in Table 2, the AWG of male Nile tilapia is lower than that of female Nile tilapia.

**Table 2.** Final body weight and survival rate of male and female Nile tilapia fed with different dietary AFB_1_ for 24 weeks.

Sex	Diet^a^	Mean final body weight (g)	Average daily gain (g/fish/day)	Average weight gain (g/fish)	Survival rate (%)
Female	1	91.67	0.37	67.15	100.00
	2	79.16	0.30	54.65	100.00
	3	68.86	0.25	44.09	100.00
	4	59.25	0.19	34.75	99.00
Male	1	91.75	0.37	67.42	100.00
	2	76.04	0.28	51.53	100.00
	3	66.99	0.24	42.66	99.93
	4	55.78	0.17	29.55	99.96
SE^b^		1.25	0.01	1.15	0.02
Dose × sex (*p*< 0.05)		0.93	<0.001	0.55	0.06
Sex effect (*p*< 0.05)		0.34	0.68	0.04	1
Dose effect (*p* < 0.05)		<0.001	<0.001	<0.001	0.14

^a^ Diet 1 = 0 μg kg^−1^ (Control), Diet 2 = 20 μg kg^−1^, Diet 3 = 200 μg kg^−1^, Diet 4 = 2000 μg kg^−1^

SE^b^ : Standard error of the difference between two means (for comparing diets within sexes) with n = 18 (individual fishes per treatment [9 males and 9 females] were measured).

**Figure 1.**
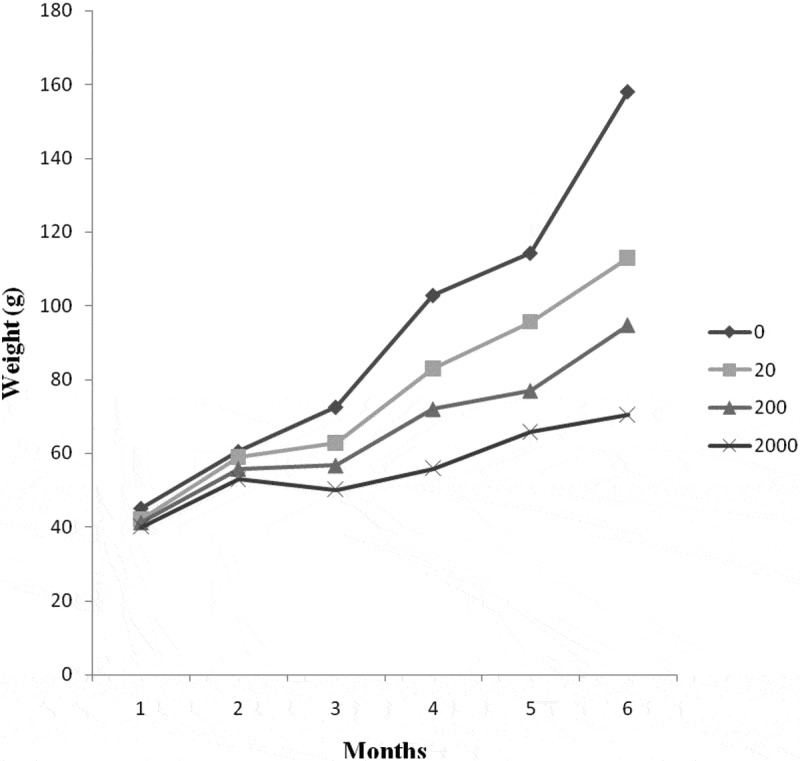
Bodyweight change of Nile tilapia fed with different dietary AFB_1_ for a duration of 24 weeks.

The effect of AFB_1_ on growth rate depended on the duration of exposure whereas, in the first 2 months, there were no differences in growth between groups with different AFB_1_ levels but by 3 months the weight gain of fish exposed to 200 and 2000 μg AFB_1_ kg^−1^ diets were lower than that of the control and 20 μg AFB_1_ kg^−1^ diet (Figure 1).

There was no significant difference in survival rate between the sexes. Survival rate was 99–100% for both male and female Nile tilapia and fish appeared healthy (Table 2).

### 3.2. Effect of AFB_1_ on reproductive hormones, egg and milt quality of Nile tilapia

Effects of aflatoxins on fecundity, egg and milt quality and sex hormones of Nile are shown in Table 3. There was no significant difference (*P <*0.05) in absolute fecundity between the control group and exposure groups. The absolute fecundity of Nile tilapia fed in control group was 435 g decreasing to 431 g in diet 4. Relative fecundity ranged from 308 ± 2.06, 310 ± 2.05, 314 ± 2.28, 550 ± 3.80 number of eggs per body weight (g) for fish fed diets 1, 2, 3 and 4, respectively. A significant difference (*P <*0.05) occurred in relative fecundity between control group and diet 4.

**Table 3.** Effects of aflatoxin on fecundity, egg and milt quality of Nile tilapia fed with different dietary AFB_1_ for 24 weeks (mean ± SE).

Measurement	Diet 1 (Control)	Diet 2	Diet 3	Diet 4
GSI for male (%)	1.88 ± 0.21^a^	1.86 ± 0.28^a^	1.69 ± 0.17^b^	0.96 ± 0.03^c^
Motility (%)	83.25 ± 1.40^a^	73.08 ± 3.44^b^	72.50 ± 1.66^b^	64.50 ± 5.66^c^
Milt count (x10^9^ sp/ml)	10.40 ± 0.98^a^	8.20 ± 0.62^b^	8.60 ± 0.57^b^	7.50 ± 0.28^c^
Milt volume (ml)	0.75 ± 0.02^a^	0.73 ± 0.01^a^	0.73 ± 0.01^a^	0.71 ± 0.02^a^
AF (total no. eggs)	435 ± 0.72^a^	433 ± 0.55^a^	434 ± 0.41^a^	431 ± 0.49^a^
RF (eggs/g bw)	308 ± 2.06^a^	310 ± 2.05^a^	314 ± 2.28^a^	550 ± 3.80^b^
Oocyte Diameter (mm)	2.80 ± 0.02^a^	2.76 ± 0.01^a^	2.78 ± 0.03^a^	2.70 ± 0.03^a^
Egg dry weight (mg)	7.02 ± 0.07^a^	7.00 ± 0.01^a^	7.01 ± 0.05^a^	7.00 ± 0.04^a^
Egg volume (mm^3^)	5.78 ± 0.06^a^	5.72 ± 0.02^a^	5.69 ± 0.03^a^	5.67 ± 0.04^a^
Total egg volume (mm^3^)	2514 ± 15.40^a^	2513 ± 23.47^a^	2481 ± 26.56^a^	2431 ± 14.40^a^
GSI for female (%)	3.30 ± 0.02^a^	3.27 ± 0.01^a^	3.26 ± 0.12^a^	2.82 ± 0.37^b^
Plasma testosterone levels (ng/ml)	2.06 ±0.47^a^	2.02 ±0.44^a^	1.97 ±0.38^a^	0.29 ±0.01^b^
Plasma oestradiol −17β (ng/ml)	3.40 ±0.09^a^	3.34 ±0.02^a^	3.32 ±0.01^a^	3.19 ±0.01^a^

Means followed by similar letter do not differ significantly (*P*< 0.05) (n = 9, individual fishes per treatment were measured), AF = absolute fecundity, RF  = relative fecundity, GSI = gonadosomatic index.

Oocyte diameter ranged from 2.80 mm in control group to 2.70 mm in diet 4, however, no significant difference was observed between control and AFB_1_ supplemented diets.

Egg volume ranged from 5.78, 5.72, 5.69 and 5.67 mm^3^ for diet 1, 2, 3 and 4, respectively, and there were no significant differences (*P <*0.05) between control group and AFB_1_ supplemented group. There was a decrease in total egg volume/ovary on fish exposed to AFB_1_ diet. Also, there were no significant differences (*P <*0.05) in egg dry weight between the control group and exposure groups.

Gonad somatic index (GSI) for female Nile tilapia values amounted 3.30% ± 0.01, 3.278% ± 0.02, 3.26% ± 0.12, 2.82% ± 0.37 for control, diet 1, diet 2, diet 3 and diet 4 respectively. There were significant differences in GSI between control group and diet 4.

Gonad somatic index (GSI) for male Nile tilapia was significantly higher in control group (1.88% ± 0.21) compared to diet 2, 3 and 4 (1.86% ± 0.28, 1.69% ± 0.17% and 0.96% ± 0.03%, respectively). The mean milt concentration among males varied from 10.4 × 10^9^ to 7.5 × 10^9^ sp ml^−1^ among the diets. The highest level of milt concentration was recorded in control group and lowest in diet 4. However, among the males having milt, mean individual milt volume did not reveal any significant differences between the diets *(P >*0.05), Table 3. Results from analysis of variance (ANOVA) showed that milt motility, milt concentration and gonad somatic index (GSI) were significantly lower in fish fed with AFB_1_ supplemented diet (Table 3).

A slight decrease in the blood serum oestradiol-17β levels were observed (3.40 ng ml^−1^ for control group and 3.19 ng ml^−1^ for diet 4), Table 3. However, no significant differences (*P* > 0.05) were observed among the four groups studied regarding blood serum oestradiol-17β in female Nile tilapia. The control group showed significantly higher concentration of testosterone in male Nile tilapia (2.06 ng ml^−1^) when compared with diet 4 (0.29 ng ml^−1^).

## 4. Discussion

### 4.1. Growth performance

The present study revealed a significant decrease in growth performances in Nile tilapia after 24 weeks period of AFB_1_ treatment. The highest growth performance was recorded in fish fed on control diet compared with the AFB_1_ supplemented diet. These results indicate that growth performance was significantly affected by AFB_1_. In general, during the 24 weeks investigation period, growth rate decreased with increased AFB_1_ concentrations. This is in line with Deng et al. [15]; El-Banna et al. [20]; Cagauan and Tayaban [21] who found that AFB_1_ exposure affected feed intake and growth performance in fish. The significant decrease in weight gain in treated groups suggests that normal digestive and nutrient absorptive functions of the intestinal epithelium were affected [22]. Deng et al. [15] and Mahfouz [23] reported that negative effects of AFB_1_ on growth could be observed only after long periods of feeding with the toxin. Similarly, in the present study, we found no difference in growth between the control group and groups with different AFB_1_ levels in the first 2 months, but by the 3 months, the weight of fish exposed to 200 and 2000 μg AFB_1_ kg^−1^ diets was lower than that of the control and 20 μg AFB_1_ kg^−1^ diets. Other studies found out that AFB_1_ did not affect growth on other fish species like Huso huso and common carp after being exposed with 100 and 2000 μg AFB_1_ kg^−1^ diets for 12 and 24 weeks, respectively [9,24]. This indicates that the effect of AFB_1_ on growth performance in fish is dependent on species, dose and duration.

We also observed lower mean weight of male Nile tilapia compared to females in all AFB_1_ supplemented diet. Female Nile tilapia fed with 2000 μg AFB_1_ kg^−1^ diet had mean body weight of 59.25 g while male Nile tilapia had 55.78 g body weight. Although, data analysis indicated no significant difference in response to AFB_1_ (*P >*0.05) on the mean body weight between male and female Nile tilapia, considering that males normally grow larger than females, this small reduction is nevertheless worth noting. On the other hand, there was a significant sex difference in response to aflatoxin B_1_ (*P <*0.05) on the AWG in the current study. Similar results were found in studies of other animal species. For example growth rates for males of pigs, birds and rats were more affected by aflatoxins exposure than their female counterparts [25–28]. More studies exposing Nile tilapia with higher concentrations of aflatoxins beyond the highest doses in the present study may determine whether male and female tilapia responds differently in growth performance and feed intake. In a previous study [29], we found aflatoxins contamination levels of up to 806 µg kg^−1^ in fish feeds and ingredients. This suggests a more serious problem that may cause low yields and economic losses in the region. Hence, controlling aflatoxin contamination throughout the fish feed value chain is necessary.

In the present study, survival rates did not differ significantly between Nile tilapia fed the control and diets containing AFB_1_ and were generally high (99–100%) for both males and females. These results are similar with Deng et al. [15]; Usanno et al. [30]; Chávez-Sánchez et al. [31] who found out that AFB_1_ does not induce mortality in tilapia fed with a diet containing more than 1000 µg kg^−1^ AFB_1_. In other studies, Tuan et al. [32] and Chávez-Sánchez et al. [31] reported that diets containing 10 and 30 mg AFB_1_ kg^−1^ respectively did not lead to mortality in Nile tilapia and concluded that tilapia can sustain a higher dose of dietary AFB_1_.

### 4.2. Effect of AFB_1_ on reproductive hormones, egg and milt quality of Nile tilapia

The most sensitive test for spermatogenesis is sperm count and it is highly correlated with fertility [33]. In fish, milt count is an important variable in influencing the fertilization of eggs. The present study found mean milt concentration varying from 10.4 × 10^9^ to 7.5 × 10^9^ sp ml^−1^ among the diets. The highest level of milt concentration was recorded in the control group and was lowest in 2000 μg AFB_1_ kg^−1^ diets. However, milt volume showed no significant differences between the diets (P > 0.05). Similar results have been reported in other animal species like Japanese quail [12], mouse [34] and rams [35]. The mechanism behind the effects of AFB_1_ on sperm count and motility in fish is not yet known but it has been described in other animal species [22,36]. Verma and Nair [37] reported that reduced succinic dehydrogenase and ATPase activity could explain the reduced sperm count and motility and increases the number of non-viable spermatozoa observed in aflatoxin-treated mice. In the present study, we monitored neither succinic dehydrogenase nor ATPase activity. However, results of the present study suggest that feeding male Nile tilapia with AFB_1_ supplemented diet for a long duration affects their milt count and may be acting in a similar manner to other animals.

The male control group showed a significantly higher concentration of testosterone (2.06 ng ml^−1^) when compared with 2000 μg AFB_1_ kg^−1^ diets (0.29 ng ml^−1^). Similar results were reported in other animal species by Bashandy et al. [7]; Kosutzka et al. [38]; Ortatatli et al. [39]; Piskac el al. [40] who found lowered plasma testosterone concentrations in rats, quail and roosters fed on AFB_1_. Decrease in testosterone levels may result in growth impairment because it has an anabolic effect on protein synthesis [36,41]. The significant decrease in the testosterone levels in the present study may be attributed to the reduction of Leydig cells and the degeneration of sertoli cells due to the presence of AFB_1_ in the diet. It is suggested by the previous study of Bbosa et al. [42] that the binding of DNA to form complexes and inhibit nucleic acid synthesis is one of the most common mechanisms for the action of AFB_1_. This mode of action may explain the direct effect of AFB_1_ on Leydig cells and Sertoli cells in the testes, and thus, the reduction of the testosterone [42,43].

Contrary to the study of Huang et al. [9] who found a reduction in absolute fecundity, egg size and volume in gibel carp fed AFB_1_ contaminated feed at 2 mg AFB_1_ kg^−1^, no significant differences in absolute fecundity, egg volume, egg dry weight and serum oestradiol-17β concentrations were found in the present study. This suggests the presence of genetic variation in sensitivity to aflatoxin among different farmed fish species which could be genetic. Further studies on intraspecies variation in sensitivity to aflatoxin would enable us to determine whether the trait is heritable and whether it can be reduced through selective breeding. This may be likely because of the physiological mechanism through which the effects of aflatoxin have been shown to operate in other species. For example in poultry, AFB_1_ significantly decreased egg production, egg volume and serum oestradiol-17β concentrations [44–46]. In another study of Washburn et al. [47] found out that AFB_1_ caused a reduction in egg size and increase in eggshell percentage, which was attributed by the fact that aflatoxins are metabolized in the liver, where the synthesis and transport of the precursors required to yolk production occur.

In this study, no significant decrease in egg size and oestradiol-17β concentrations was observed between control and exposed group. However, the result shows a trend towards decreasing levels of egg size and oestradiol-17β concentrations with increase in AFB_1_ concentrations in the diet.

In addition, in the present study, a significant decrease in GSI was observed only in higher‐doses (2000 μg AFB_1_ kg^−1^) in female Nile tilapia. On the other hand, in male Nile tilapia a significant decrease in GSI was observed in 200 and 2000 μg AFB_1_ kg^−1^ diets suggesting sex-linked variation in levels of sensitivity among Nile tilapia.

## 5. Conclusion

Prolonged feeding of Nile tilapia with high levels of AFB_1_ for 24 weeks reduced body weight of Nile tilapia in the present study. Males of Nile tilapia were more susceptible to dietary exposure of AFB_1_ than their female counterparts as exhibited by the effects on different reproductive parameters. On the contrary, absolute fecundity, egg weight and size, oocyte diameters and oestradiol-17β of female Nile tilapia were not affected by AFB_1_. This indicates the necessity of improved management practices during preparation and handling of fish feeds as this may affect pond yields and production of fish seed in hatcheries. Studies on the existence of genetic variation in sensitivity to aflatoxin within species may point to possibility for control of aflatoxin effects through genetic means.
